# Supplementary material for: Floral transcriptomes reveal gene networks in pineapple floral growth and fruit development
Source: Commun Biol. 2020 Sep 10;3:500. doi: 10.1038/s42003-020-01235-2 (PMC7483743; doi:10.1038/s42003-020-01235-2)
Supplement: Supplementary file 1 — Supplementary Information [file 42003_2020_1235_MOESM1_ESM.pdf]

Supplemental Figure 1

A

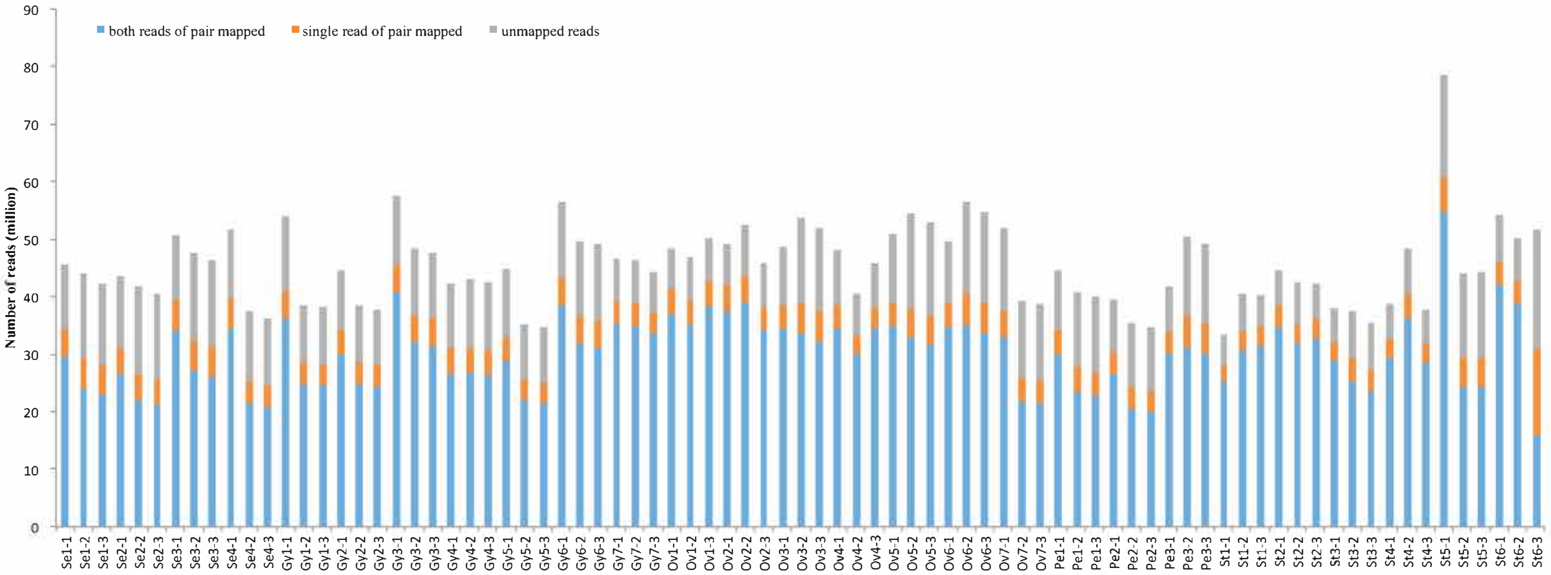

B

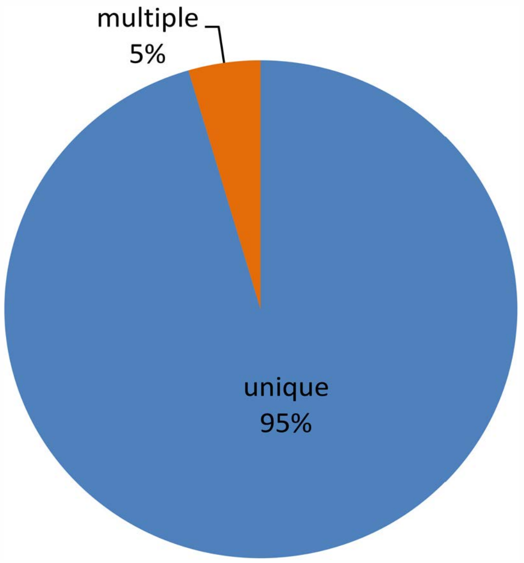

Supplementary Figure 1. Statistics for the mapping of RNA-seq reads to the pineapple reference genome assembly

A For each sample and biological replicate, the number of RNA-seq read pairs that fell into the following alignment categories: both reads aligned (light blue), one read aligned (orange) and unmapped (gray). The pineapple reference genome assembly was used for mapping

B. Percentage of uniquely mapped reads (exactly one mapping location; blue) and reads with multiple mapping locations with identical alignment scores (orange)

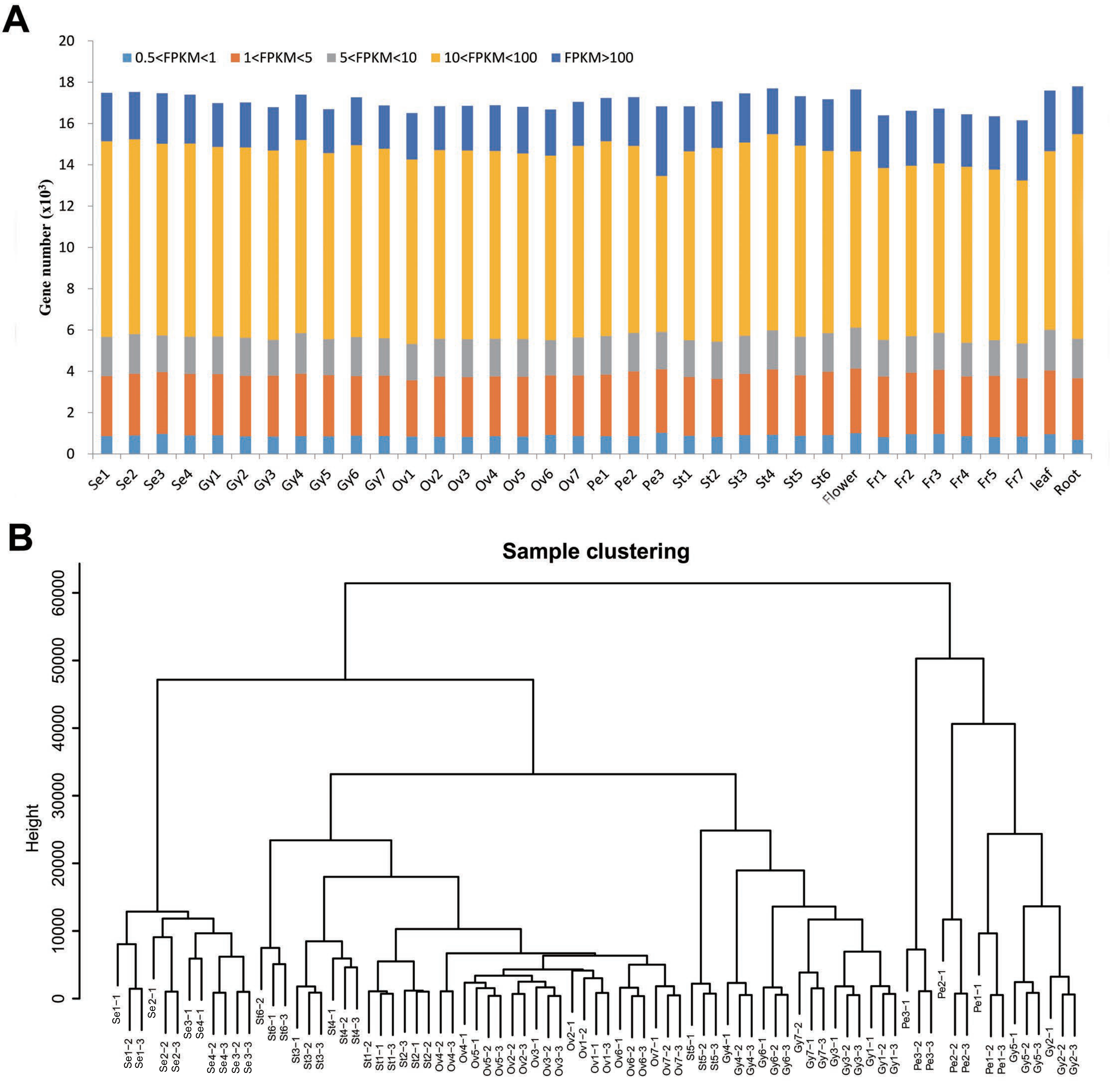

Supplementary Figure 2. Global analysis of pineapple RNA-seq data.

A. The distribution of genome-wide gene transcription levels derived from the pineapple RNA-seq data.

B. Hierarchical agglomerative sample clustering of the floral samples. Note that three biological replicates are clustered together, indicating the reproducibility of the data.

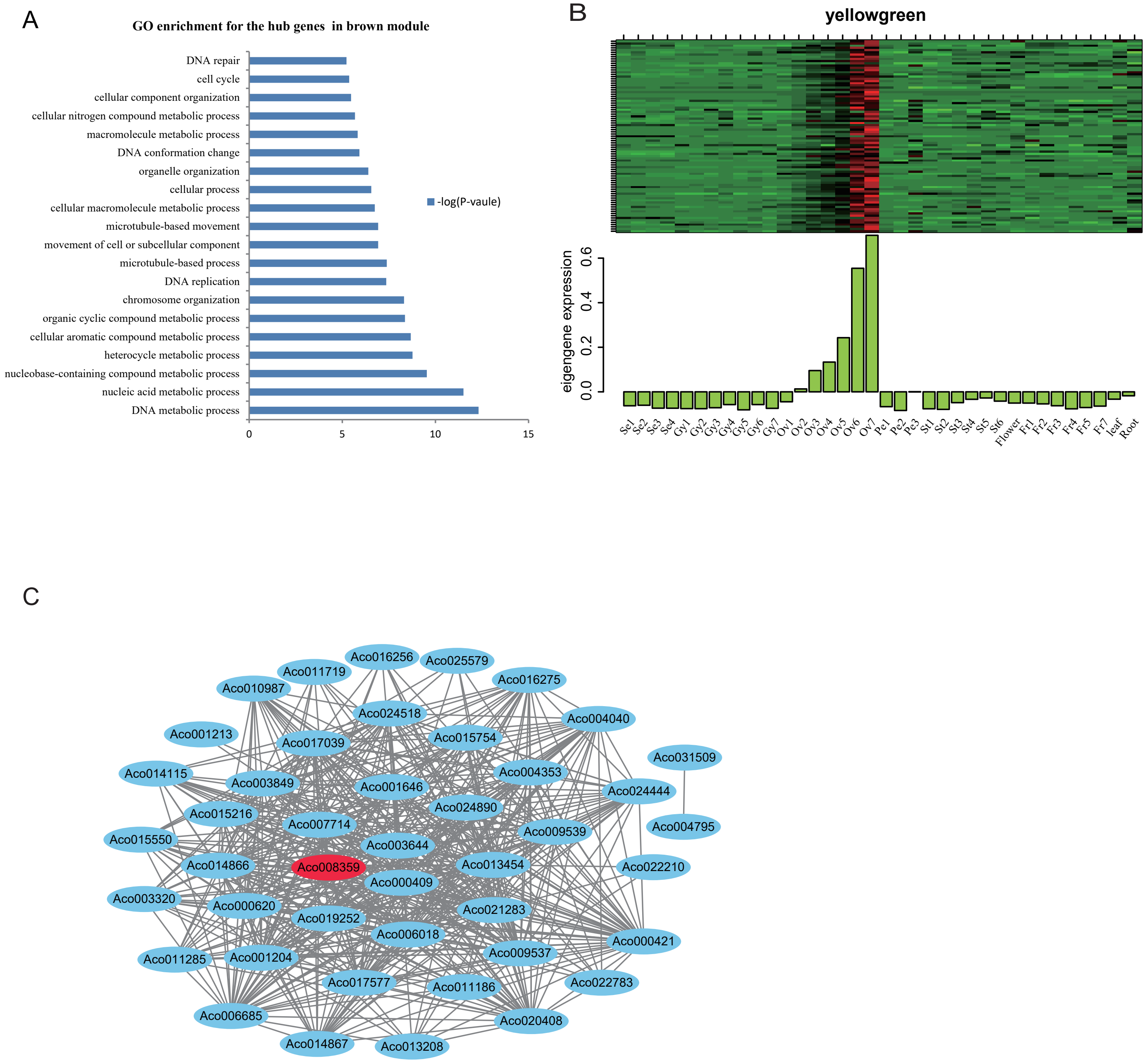

Supplementary Figure 3. Genes and network diagram of the yellowgreen module.
A. GO enrichment for the hub genes in brown module.
B. Eigengene expression profile for the yellowgreen module in different tissues. Top panel: expression heatmap showing the relative FPKM of each gene from the yellowgreen module. Bottom panel: the x axis indicates the samples, and the y axis indicates the log2 “relative FPKM values” of the module eigengene.
C. Correlation network of the yellowgreen module. The Cytoscape-generated diagram includes 44 genes with edge weights greater than 0.3. The red oval indicates a transcription factor gene.

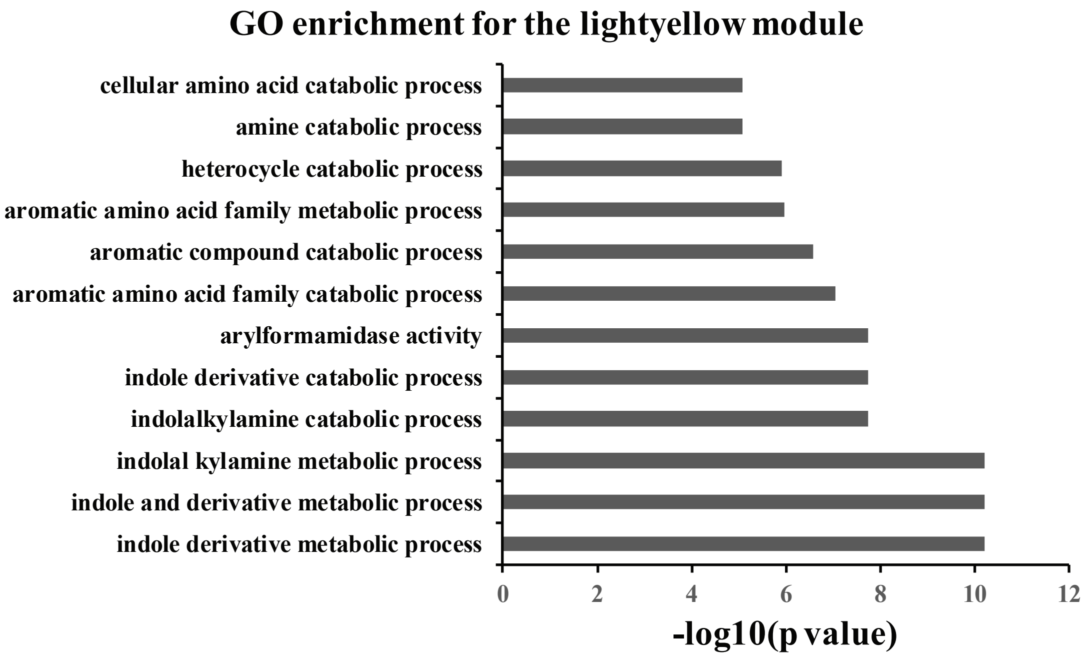

Supplementary Figure 4. GO enrichment for the lightyellow module.

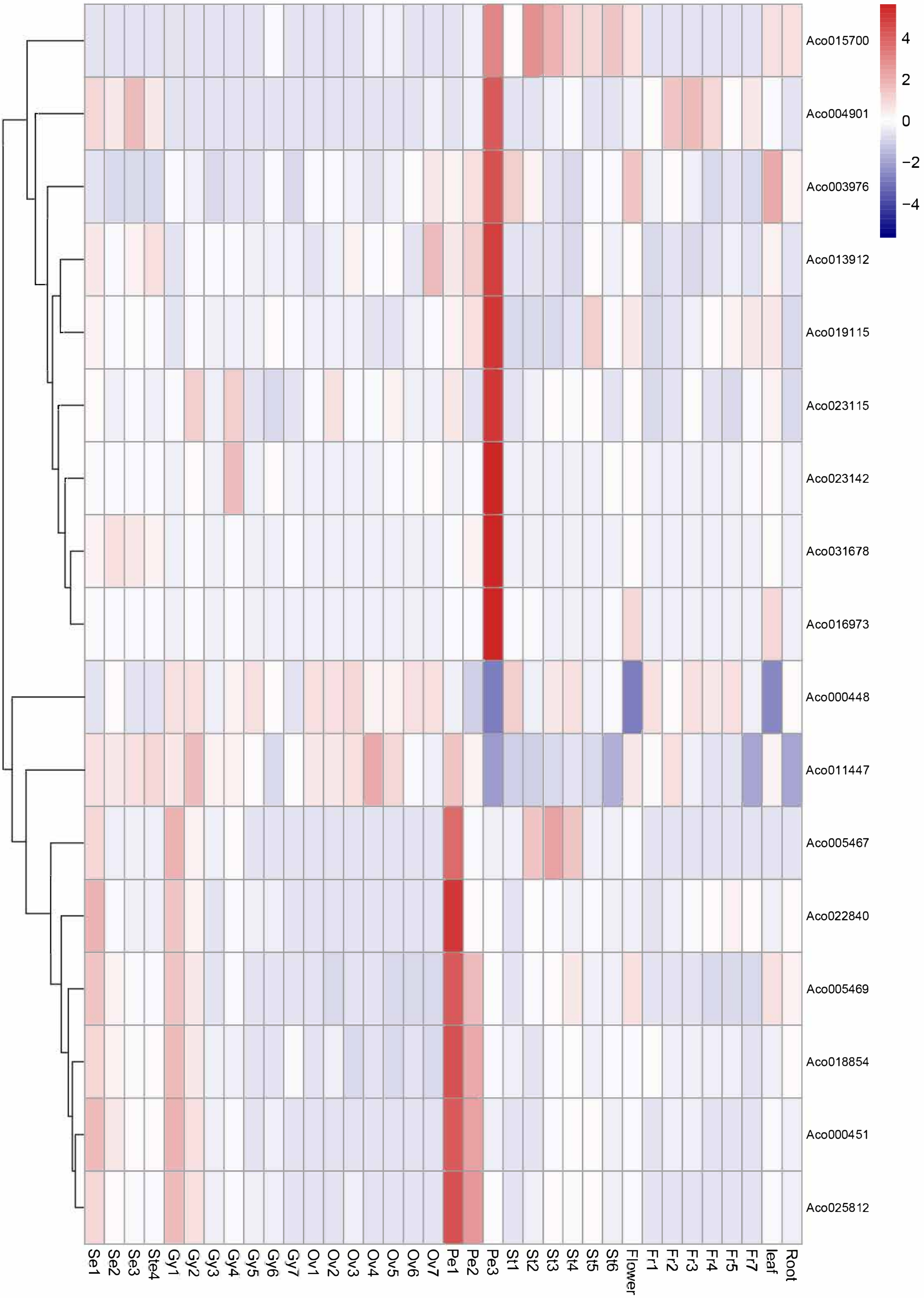

Supplementary Figure 5. Heatmap showing the log2 "relative FPKM values" of the six Petal 1-associated subtilase genes and 11 Petal 3-associated subtilase genes in different samples.

A

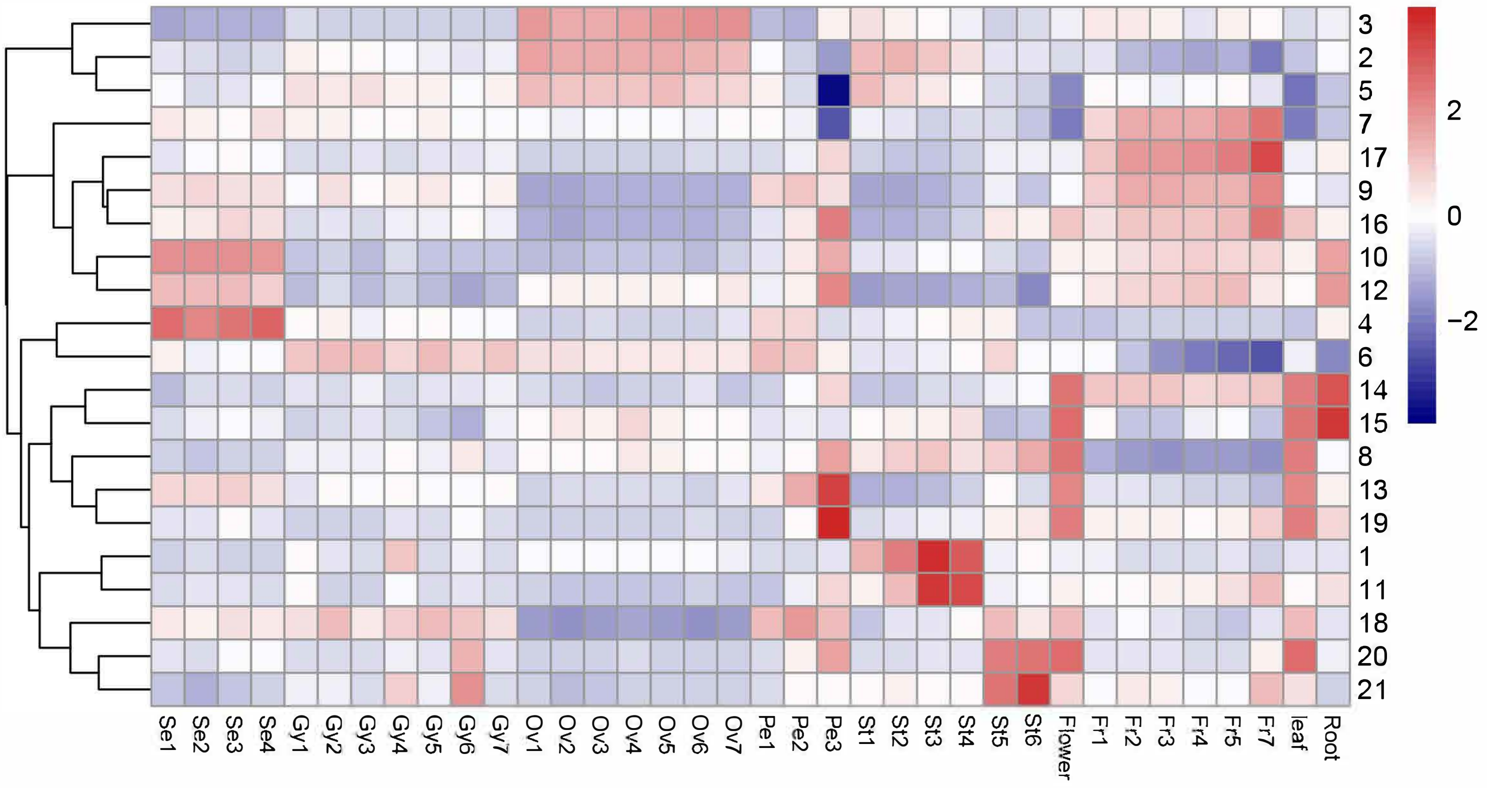

B

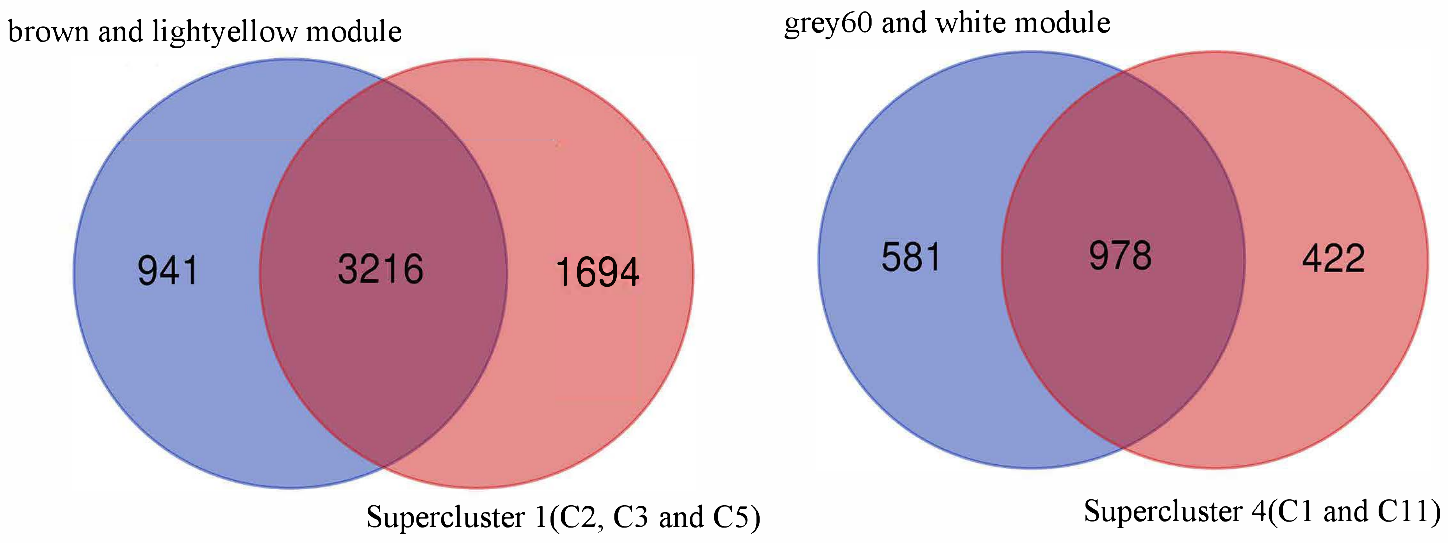

Supplementary Figure 6.

A Twenty-one K-means clusters of19,832 genes with distinct tissue- and stage-specific expression patterns

B. Venn diagram showing the overlap ovule associated genes and stamen associated genes between WGCNA and K-means.

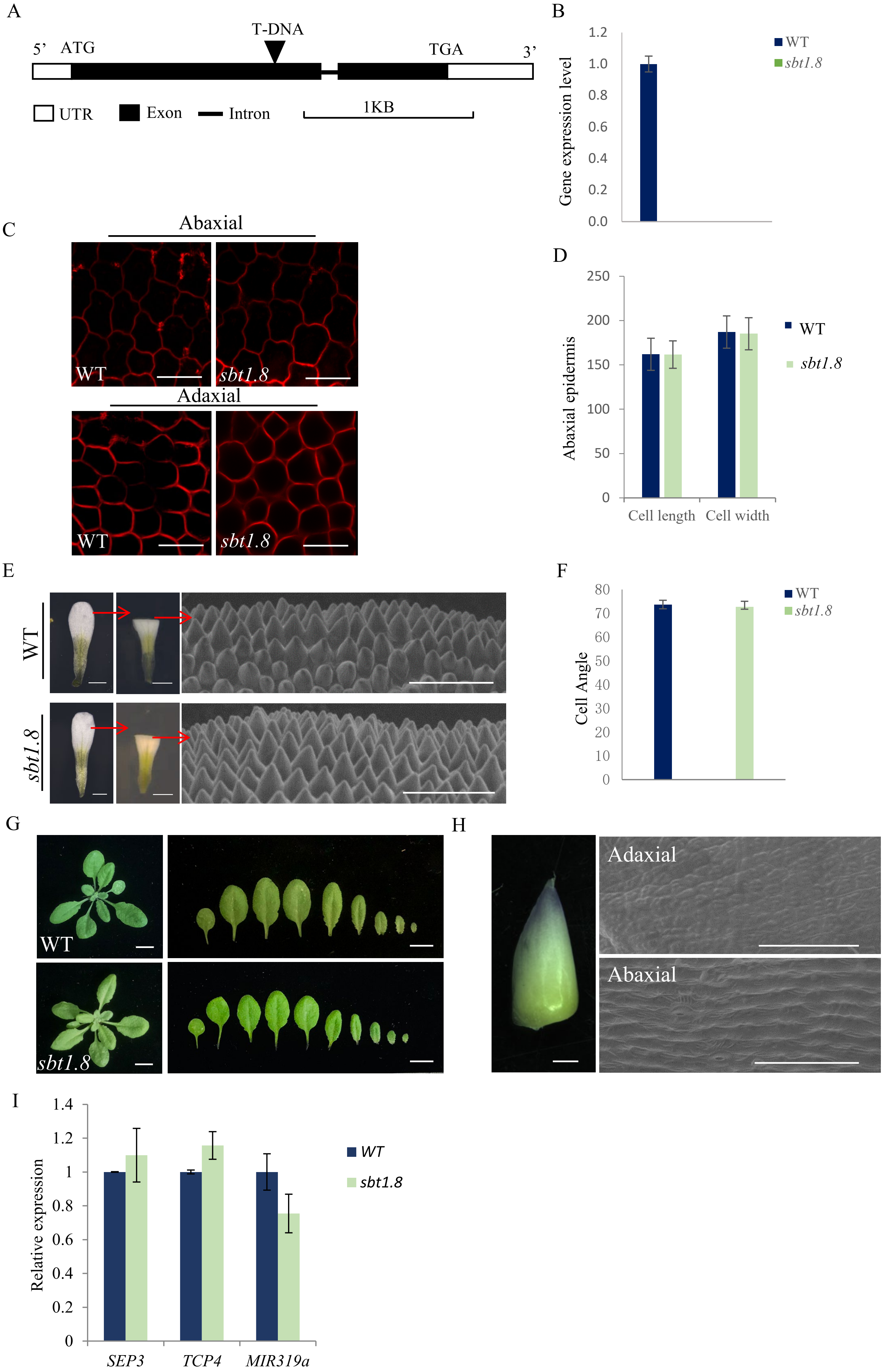
A
T-DNA
5' ATG
TGA 3'
UTR Exon Intron
1KB
B
Gene expression level
1.2
1.0
0.8
0.6
0.4
0.2
0.0
WT
sbt1.8
C
Abaxial
WT
sbt1.8
Adaxial
WT
sbt1.8
D
Abaxial epidermis
250
200
150
100
50
0
Cell length
Cell width
WT
sbt1.8
E
WT
sbt1.8
F
Cell Angle
80
70
60
50
40
30
20
10
0
WT
sbt1.8
G
WT
sbt1.8
H
Adaxial
Abaxial
I
Relative expression
1.4
1.2
1
0.8
0.6
0.4
0.2
0
SEP3
TCP4
MIR319a
WT
sbt1.8

Supplementary Figure 7. Phenotypic characterization of the *sbt1.8* mutant.

A. *AtSBT1.8* gene diagram including the Salk_020799 T-DNA insertion site.
B. *AtSBT1.8* expression level in WT and *sbt1.8*.

C-D. Petal blade abaxial and adaxial cell phenotypes and cell size quantification in WT and*sbt1.8.* Bar = 100 µm.
E-F . Petal blade adaxial conical cell phenotype analyzed by scanning electron microscopy (SEM) and quantification of the conical cell angle in WT and *sbt1.8*. Bar = 1 mm in the stereomicroscope images and 100 µm in the SEM images.
G. The leaf phenotype of WT and the sbt1.8 mutant. Bar = 1 mm.
H. Pineapple petal blade adaxial cell phenotype analyzed by SEM. Bar = 1 mm in the stereomicroscope image and 100 µm in the SEM images.
I. The expression of SEP3, TCP4 and MIR319a in WT and sbt1.8 mutant.

Supplementary Table 1. Ten transcriptional factors (TFs) among the lightyellow hub genes.

ID	kME	P-value	Description	TF	Ortholog gene
Aco000069	0.927	5.09E-16	WRKY family transcription factor family protein	WRKY	AT4G26640/AtWRKY20
Aco002567	0.902	6.08E-14	WRKY DNA-binding protein 48	WRKY	AT5G49520/AtWRKY48
Aco018555	0.912	l.05E-14	NAC domain containing protein 17	NAC	AT1G34190/ AtNAC017
Aco008183	0.975	6.91E-24	Homeobox protein knotted-I-like protein	HB:TALE	AT1G23380/AtKNAT6
Aco012803	0.915	6.21E-15	Homeobox transcription factor KN4	HB:TALE	AT4G08150/ AtKNAT1
Aco015871	0.967	l.l lE-21	Homeobox protein knotted-I-like protein	HB:TALE	AT5G25220/ AtKNAT3
Aco001167	0.902	6.19E-14	bZIP transcription factor family	bZIP	AT4G00120/ AtIND1
Aco005342	0.906	2.80E-14	Transcription factor bHLH144	bHLH	AT1G29950/ AtSACL3
Aco009511	0.941	l.31E-17	Ethylene-responsive transcription factor	AP2/ERF:ERF	AT1G53910/ AtERF74
Aco021063	0.942	9.98E-18	Ethylene-responsive transcription factor	AP2/ERF:ERF	AT3G14230/ AtRAP2.2
