# Supplementary material for: Floral transcriptomes reveal gene networks in pineapple floral growth and fruit development
Source: Commun Biol. 2020 Sep 10;3:500. doi: 10.1038/s42003-020-01235-2 (PMC7483743; doi:10.1038/s42003-020-01235-2)
Supplement: Supplementary file 2 — Description of Additional Supplementary Files [file 42003_2020_1235_MOESM2_ESM.pdf]

Descriptions of Additional Supplementary Files

Supplementary Data 1. Mapping statistics for the alignment of reads to the pineapple reference genome for each library.

Supplementary Data 2. The distribution of genes in each sample according to their expression levels.

Supplementary Data 3. Gene information for each branch leaf in the hierarchical clustering dendrogram.

Supplementary Data 4. The correlation of each tissue sample to the identified modules.

Supplementary Data 5. Highly enriched GO categories in each module.

Supplementary Table 6. Detailed information for the hub genes.

Supplementary Data 7. The expression patterns of transcription factor (TF) genes in the 21 K-means clusters.

Supplementary Data 8. Gene information and the ortholog genes in Arabidopsis and rice for TF genes in supercluster 1- 7.

Supplementary Data 9. Expression values of A, B, C, and E class genes in pineapple floral and vegetative tissues.

Supplementary Data 10. Primers used this study.
